# How Functional Genomics Can Keep Pace With VUS Identification

**DOI:** 10.3389/fcvm.2022.900431

**Published:** 2022-07-04

**Authors:** Corey L. Anderson, Saba Munawar, Louise Reilly, Timothy J. Kamp, Craig T. January, Brian P. Delisle, Lee L. Eckhardt

**Affiliations:** ^1^Cellular and Molecular Arrythmias Program, Division of Cardiovascular Medicine, Department of Medicine, University of Wisconsin-Madison, Madison, WI, United States; ^2^Department of Physiology, University of Kentucky College of Medicine, Lexington, KY, United States

**Keywords:** functional genomics, VUS classification, cardiac genetics, high through put screening, inherited arrhythmia

## Abstract

Over the last two decades, an exponentially expanding number of genetic variants have been identified associated with inherited cardiac conditions. These tremendous gains also present challenges in deciphering the clinical relevance of unclassified variants or variants of uncertain significance (VUS). This review provides an overview of the advancements (and challenges) in functional and computational approaches to characterize variants and help keep pace with VUS identification related to inherited heart diseases.

## Introduction

**Figure 1:**
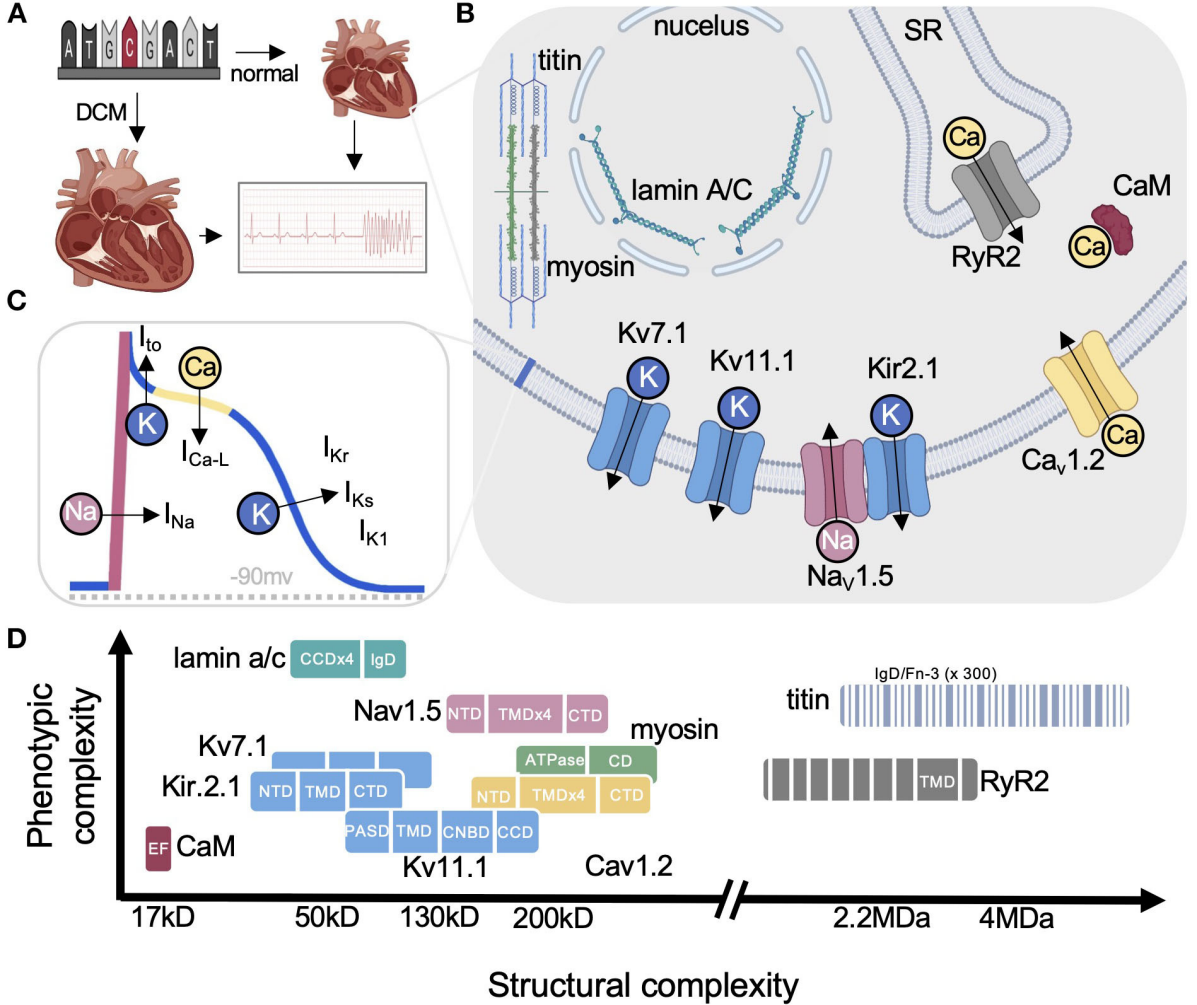
Cardiac disease proteins highlighted in this review. **(A)** Arrhythmias can result from structurally abnormal hearts (e.g., DCM) or normal hearts with electrical abnormalities (e.g., LQTS). **(B)** Simplified cartoon highlighting each protein and their primary functions. **(C)** Ventricular action potential showing the major ionic currents. **(D)** Graph illustrating the range in size for each protein discussed on the x-axis from small calmodulin to the the largest human protein titin comprised of hundreds of small soluble domains (eg., IgD). The y-axis shows the relative pleiotropy for each gene (i.e., relative number of diseases associated with each). For example, CaM has only been linked to LQTS while lamin underlies over a dozen diseases. Nav1.5 on the other hand is a good example of pleiotropy among the ion channels reviewed by Cerrone et al. (3) with lamin being the most pleiotropic. DCM, dilated cardiomyopathy; SR, sarcoplasmic reticulum; RYR2, ryanodine receptor 2; CaM, calmodulin; CCD, coiled-coil domain; CD, converter domain; IgD, immunoglobulin-like domain; NTD, FN-3, fibronectin type III-like; N-terminal domain; TMD, transmembrane domain; PASD, PerArntSim domain; CNBD, cyclic nucleotide-binding homology domain. Some figure panels created with BioRender.com.

**Figure 2:**
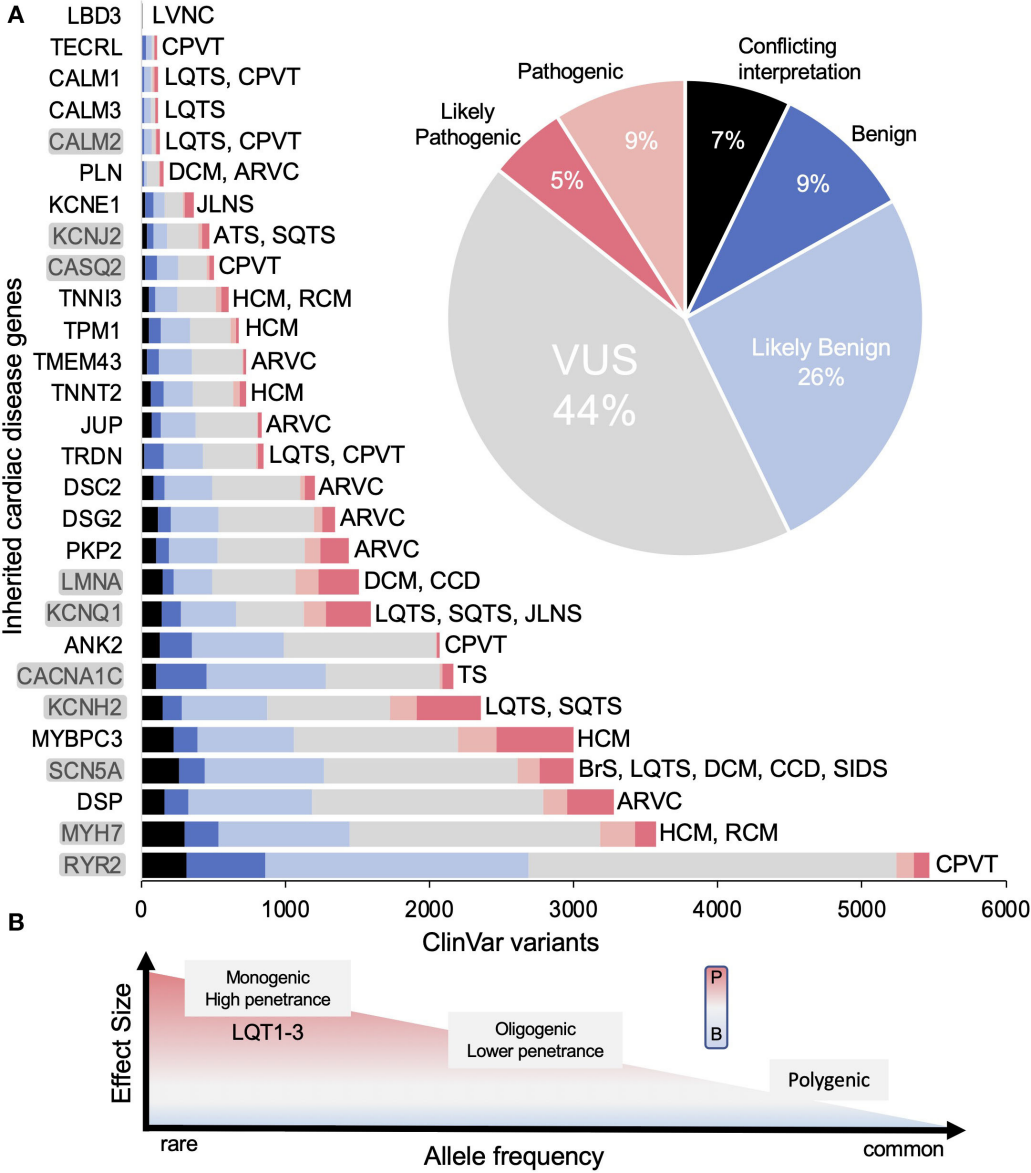
Genes associated with inherited cardiac arrhythmias. **(A)** The total number of variants for each gene in ClinVar and their classifications [conflicting interpretation (CI), likely benign (LB), benign (B), variant of uncertain significance (VUS), likely pathogenic (LP) and pathogenic (P)] as of June 2022. Inset shows relative percentages for all genes combined. Disease associations are not comprehensive (e.g., LMNA is linked to many non-cardiac diseases). Genes reviewed here shaded gray. **(B)** Graph illustrating the range of effects variants can have toward pathogenicity. Figure adapted from Ingles et al. (8). Disease abbreviations: CPVT, catecholaminergic polymorphic ventricular tachycardia; HCM, hypertrophic cardiomyopathy; RCM, restrictive cardiomyopathy; ARVC, arrhythmogenic ventricular cardiomyopathy; BrS, Brugada syndrome; LQTS, long QT syndrome; DCM, dilated cardiomyopathy; CCD, cardiac conduction disease; SIDS, sudden infant death syndrome; SQTS, short QT syndrome; TS, Timothy syndrome; JLNS, Jeverell and Lange-Nielsen syndrome; ATS, Andersen-Tawil syndrome; LVNC, left ventricular non-compaction cardiomyopathy.

Genetic mutations can affect the heart's structure (1) or electrical system (2), which can cause a variety of life-threatening arrhythmias (Figure 1A). Since the discovery of the first Long QT Syndrome (LQTS)- associated genes; KCNQ1 encoding Kv7.1 (LQT1) (4), KCNH2 encoding Kv11.1 (LQT2) (5), SCN5A encoding Na_v_1.5 (LQT3) (6) and cardiomyopathy-associated MYH7 gene encoding β-myosin heavy chain (7) in the early 1990s, many other ion channels (e.g., KCNJ2 encoding Kir2.1) and functionally diverse proteins have been implicated in a variety of other clinical phenotypes (see Figure 2A for a list of common ones) (9). Initially, these diseases were collectively thought to be predominantly LQT-associated and Mendelian in nature. However, recent insights from large sequencing initiatives (10) and phenotype data from electronic medical records are challenging this view (8, 11–13) (Figure 2B). This insight is further amplified by a recent study of the large population-based United Kingdom Biobank (UKBB) (14) and Trans-OMICs for Precision Medicine (TOPMed) biobank (15) showing LQTS to be a more complex disease with most genetic factors unaccounted for (3, 16).

Advances in sequencing technology have revolutionized clinical and translational cardiology, yet enthusiasm wanes in light of the deluge of uncharacterized variants now reported in ClinVar (17). To give a sense of scale of this problem, titin, the largest human protein has over 7,000 VUS alone, which are linked to at least four cardiac diseases including familial cardiomyopathies DCM (Dilated Cardiomyopathy, most common), RCM (Restrictive Cardiomyopathy) and HCM (Hypertrophic Cardiomyopathy) as well as ACM (Arrhythmogenic Cardiomyopathy) (18). Of the remaining more common arrhythmia-linked genes, ~50% are classified as VUS or conflicting interpretation in ClinVar. The magnitude of this problem is highlighted in Figure 2A showing that the identification of coding variants has far outpaced our ability to correctly classify variants. From a clinical standpoint, identification of VUS creates substantial barriers as these are not clinically actionable and misinterpretation has serious ramifications for sudden cardiac death assessment of the patient and their family (19–23). The importance of physiologic and functional analysis for variant classification has been emphasized, yet contemporary methods are cumbersome (time and resources) decreasing efficiency in unraveling the arrhythmic risk associated with genetic variants.

**Figure 3:**
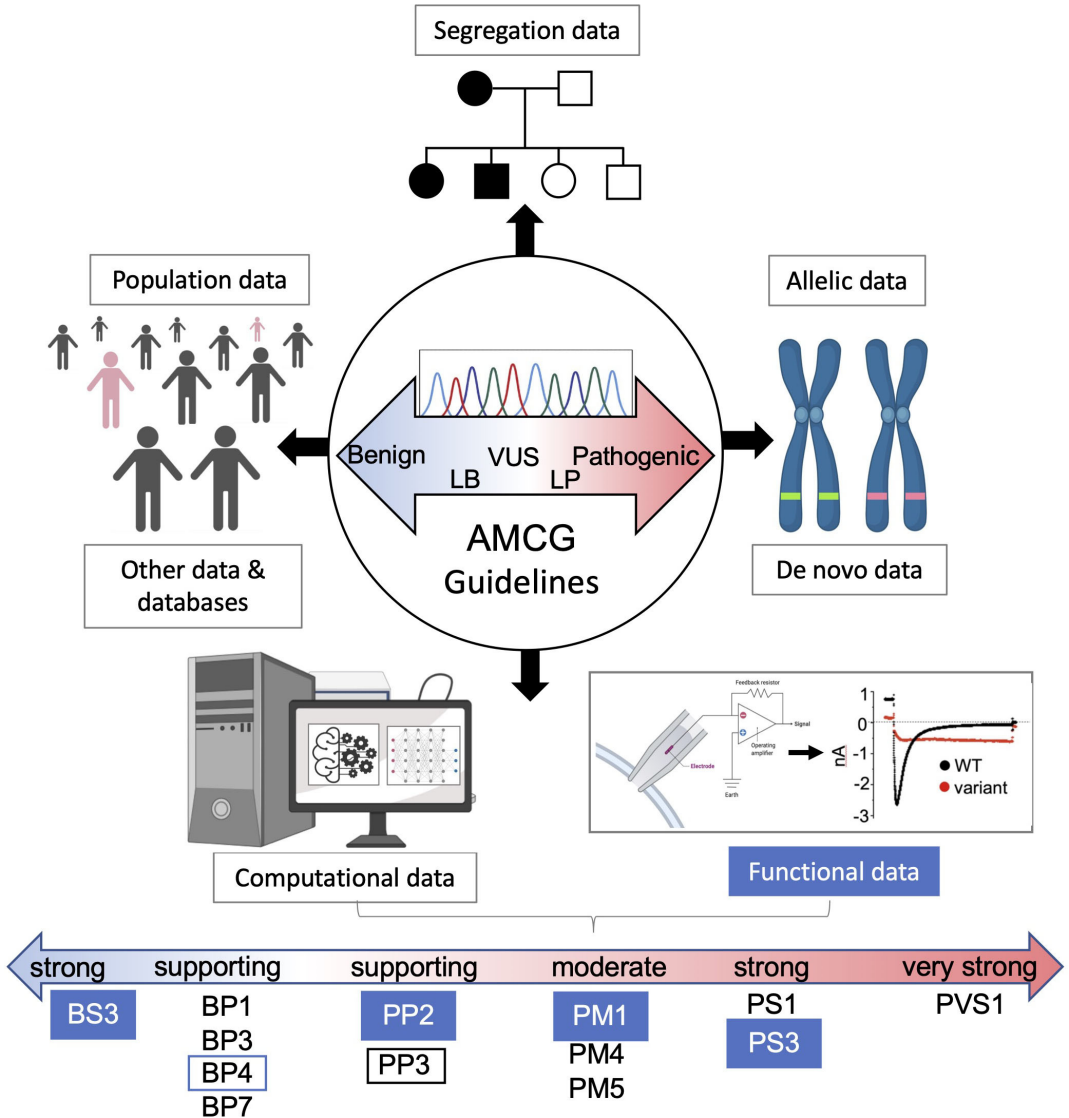
AMCG Guidelines for variant interpretation. A brief overview of the eight lines of supporting evidence for evaluating variants of uncertain significance. Numerous benign and pathogenic supporting computational and functional levels are shown for each approach with functional evidence filled blue. The lines of evidence described by Richards et al. (27) in the guidelines are as follows: BS3/PS3, not damaging/damaging functional studies; BP1, missense variant in a gene with primarily truncating variants; BP3, indels in repeat region without known function; BP4/PP3, multiple lines of computational evidence showing no damaging effects/damaging affects; BP7, non-splicing, silent variant; PP2, variant in gene where pathogenic variants are common and benign variants are few; PM1, variant hotspot without benign variants; PM4, protein length changing variant; PM5, variant at residue with other pathogenic variants; PS1, amino acid change same as other pathogenic variant(s); PVS1, null variant in a pathogenic gene with known LOF.

However, new technologies are primed to help close the gap in VUS interpretation including higher throughput functional methods such as automated patch clamp (24), deep mutational scanning (DMS) (25) and a myriad of *in silico* tools (26). Such data is useful for supporting evidence across the 8 categories utilized for variant pathogenicity by the American College of Medical Genetics and Genomics (ACMG) standard (27). This is used to semi-quantitatively designate a variant as benign (B), likely benign (LB), uncertain significance (VUS), likely pathogenic (LP) and pathogenic (P) (illustrated in Figure 3). Ultimately, accurate assessment of VUS requires knowledgeable professionals conducting a review of these factors from the literature using these ACMG guidelines for interpretation, all under the standardized umbrella of ClinGen (28). These guidelines have continually undergone refinement (29–31) and further gene or disease specific revision is needed (32) as big data grows. With many excellent reviews already covering various aspects of these inherited cardiac conditions (33, 34) and methods (35, 36) this focuses on recent functional and computational advances to help interpret cardiac disease missense VUS using several ion channels and structural proteins as examples (Figure 1B).

## High Throughput Functional Assays

**Figure 4:**
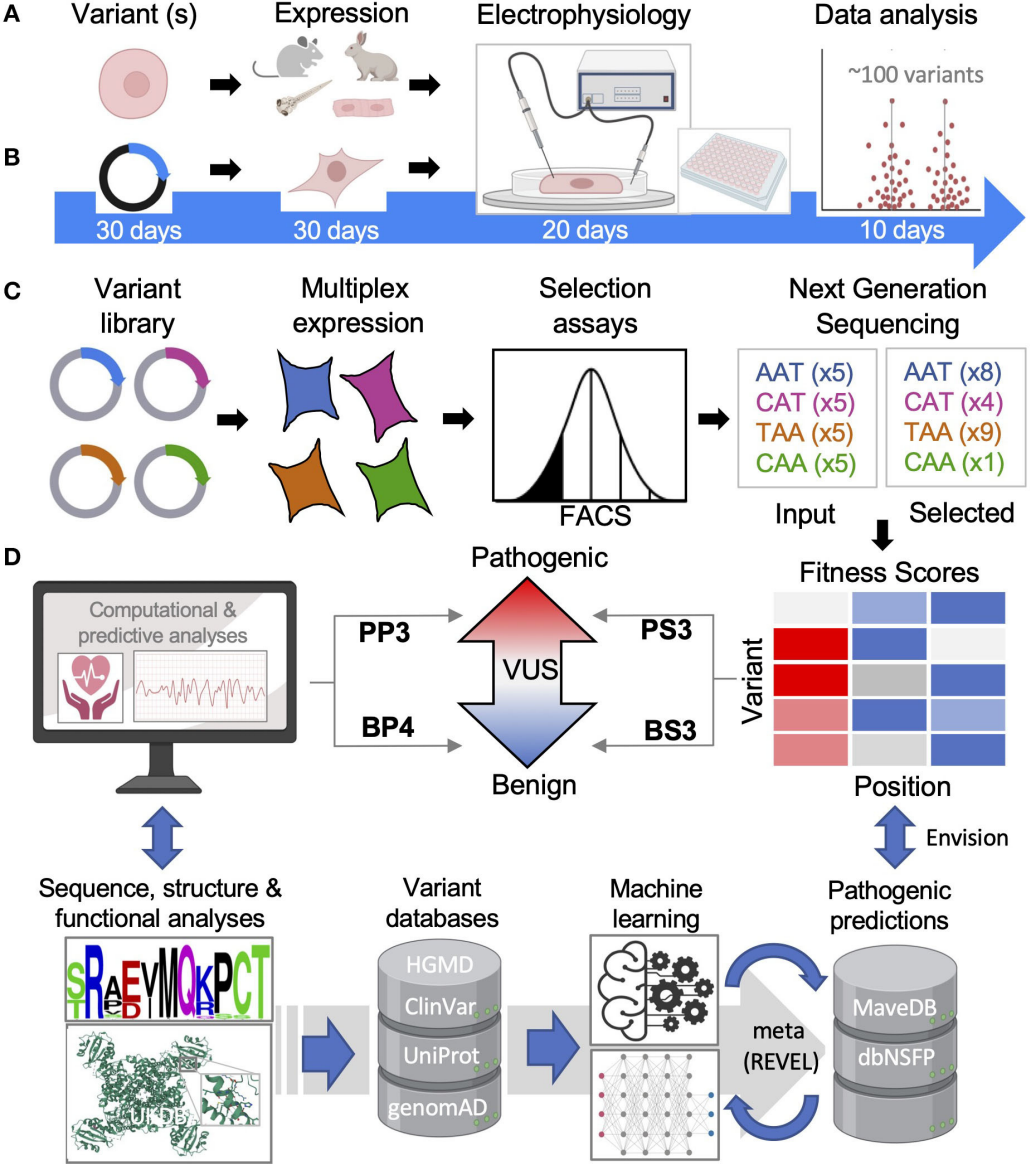
Functional and computational methods of inherited cardiac arrhythmias. **(A)** Animal and iPS-CM functional models will remain important but are low throughput. **(B)** Overexpression systems are higher throughput especially when automated with functional results for ~100 variants reported in less than two months' time by Ng et al. (38). **(C)** Multiple assays of variant effects (MAVEs), while largely proxy assays, are magnitudes higher throughput allowing for comprehensive variant analyses. **(D)** Tools to predict a VUS can be simple and rule-based relying on sequence and/or structural data or, more commonly, machine learning methods are employed. FACS, fluorescence-activated cell sorting; HGMD, Human Gene Mutation Database; UniProt, Universal Protein Resource; genomAD, Genome Aggregation Database; REVEL, Rare Exome Variant Ensemble Learner; dbNSFP, Database for Functional Predictions of non-synonymous SNPs.

Well established functional tests are considered strong evidence for pathogenic classification given they are sufficiently validated with a proper number of positive and negative controls (37). Two significant developments in particular, automated patch (24) and Deep Mutational Scanning [DMS, also called multiplex assays of variant effects (MAVE)] (25) have recently made large strides toward large-scale characterization of ion channelopathy VUS. Briefly, we summarize each technology below (see Figure 4 for context) and then highlight how each has been applied for several specific cardiac proteins.

### HTP Electrophysiology

For ion channelopathies and other arrhythmogenic diseases, changes in cellular action potentials are arguably the best cellular assessment of pathogenicity and a variety of *in vivo* models have been utilized each with advantages and disadvantages (39) (Figure 4A). Interestingly, zebrafish have shown to be a promising higher-throughput approach, demonstrating high accuracy when 49 LQT2-associated variants were benchmarked against known benign and pathogenic variants (40). However, these *in vivo* models lack a native-like environment with differences in ion channel expression in mice and a two-chamber heart in zebrafish for example (41). Human induced-pluripotent stem cell-derived cardiomyocytes (iPS-CMs) have proven very useful for modeling inherited cardiac diseases (42), even identifying genetic modifiers that can influence a specific variant's effect (43). Throughput of iPS-CM characterization can also be greatly improved with the use of multi-electrode arrays as a proxy for measuring action potentials (44). There remain limitations with iPS-CMs including their electrical and metabolic maturation (45) as well as generating cells lines in large quantities. Advanced culturing techniques and co-culturing with cardiac fibroblasts (46) and other maturation techniques (47) may help address some of these issues and assist with consistency. Further, a recent technique was reported to immortalize primary atrial CMs, which could greatly scale iPS-CM production if transferrable (48).

Measuring ionic currents from overexpression models like HEK 293 cells is the main workhorse in ion channelopathy variant characterization. Advancements in automation have dramatically increased throughput (Figure 4B), with a recent study analyzing ~100 variants within 2 months', making comprehensive functional analysis of all known VUS within reach (38). For example, KCNH2 has ~700 missense VUS or conflicting interpretations that could all be assessed using this method in about one year by one dedicated lab (Figure 2A). The combination of ionic current characterization with automation using planar patch makes it conceivable that the function of all reported ion channelopathy VUS can be characterized within the coming years, and we review recent proof-of-principle studies toward that goal below.

### Deep Mutational Scanning

When a protein's function is multifactorial or diverse, one powerful approach for characterizing variants is the development of deep mutational scanning (DMS). While the signal for pathogenicity is conceptually simpler for ion channels (current vs. changes in current), other proteins with diverse functionalities also have thousands of VUS requiring other HTP approaches (Figures 1A, 2). Further, variants may impact pathogenicity through multiple mechanisms, and so assays that target different mechanisms will also be valuable for personalized medicine. Targeted correction of trafficking defective LQT2 variants is a recent example of how this can be used. Since the discovery of several mechanistic classes of LQT2 variants, first described in the January Lab 25 years ago (49), feasible correction for class 2 trafficking defective variants has recently been realized in patient iPS-CM cell lines (50) and validated in two LQT2 patients (51).

DMS is enabled by expansive parallel mutagenesis technology (52). These parallel LoF analyses or MAVES allow for truly HTP analyses of all possible variants of a protein of interest (25, 53). The power of DMS hinges on suitable multiplexable assays. As illustrated in Figure 4, using an ion channel as an example, a comprehensive library of variant plasmids (modified with an extracellular tag for cell surface detection) is overexpressed in a suitable cell model (HEK 293 cells) and a phenotype is selected for (cell surface expression by FACS) (Figure 4C). Cell fractions are collected and sequenced using next generation sequencing to determine the frequency of each variant and, ultimately a functional score reflective of cell surface expression is calculated. DMS is a proactive approach to variant identification as all possible variants are simultaneously characterized and can be publicly available in functional databases (i.e., MaveDB) (54). Thus, as new variants are identified clinically, the functional properties will have been characterized and can be referenced rather than reactively studied. This growing database will serve as a rich source of functional data providing unprecedented molecular insights and potential toward interpreting VUS within ACMG guidelines (55) (Figure 4D).

Finally, these methods are powerful for characterizing coding variants like missense (the most common type), but other types such as synonymous and non-coding variants can also be prevalently associated with cardiac disease (56–58). While not the focus of this review, HTP functional advances for these types of variants have also been made such as massively parallel reporter assays (MRPAs), which can assess potential splice-site variants (59) and regulatory regions (36) in HTP. Applying these techniques should be invaluable toward interpreting potential cardiac splice-altering variants and improve our understanding of inherited cardiac arrhythmias.

### Specific Gene Examples of HTP Analysis

Genes, associated with arrhythmogenic disease that have a high number of VUS (Figure 2A) are particularly suited for HTP characterization. Several genes that encode for Nav1.5, Kv7.1 and Kv11.1 have recently been functionally studied using automated patch clamp. Various types of DMS have also been reported for Nav1.5, Kv11.1, Kir2.1 and calmodulin. We also discuss a few other protein classes including Ca handling (L-type calcium channel, ryanodine receptor, calmodulin), as well two filament proteins involved in numerous cellular processes (titin and lamin A/C), to highlight some challenges for broad HTP functional characterization of proteins and suggest potential approaches.

## SCN5A/Nav1.5

Nav1.5, the cardiac voltage-gated sodium channel encoded by SCN5A, is responsible for the cardiac action potential's rapid inward sodium current (*I*_*Na*_) (Figures 1B,C) and shares many common structural features with voltage-gated potassium and calcium channels (i.e., tetrameric with voltage-sensor, pore, and specialized intracellular domains) (Figure 1D). However, variants in SCN5A1 are more pleiotropic, causing several electrical disorders including the loss-of-function (LoF) Brugada Syndrome (BrS) (most common), and gain-of-function (GoF) LQTS, as well as cardiomyopathy (60) (Figure 1D). ClinVar lists over 1100 missense VUS or with conflicting interpretation with numerous LoF mechanisms (i.e., loss of sodium conductivity or channel availability) described through patch clamp analyses (61).

Glazer and co-authors recently demonstrated automated patch clamp as a reliable higher throughput approach for assessing Nav1.5 VUS (62). After replicating published electrophysiological data for 10 variants and showing no false positives for 10 purported benign controls, they reported a range of peak current densities for 63 other variants and suggested pathogenic reclassifications for all except 12. Extrapolating their patch clamp data has the potential to reduce the total Nav1.5 VUS functional burden by at least 75%. This assay was further validated in a study of 22 Nav1.5 variants showing patch clamp data to be an excellent predictor of pathogenicity and lethal cardiac arrhythmias in Brugada Syndrome (63).

As a complementary approach, Glazer and co-authors also reported the clever use of a DMS by adapting a three-drug cytotoxicity assay to assess the function of all possible GoF and LoF Nav1.5 variants in a small region of the S4 voltage sensor (64). In this assay, the agonists veratridine and brevetoxin are used to open the channel causing a constant influx of Na. Concurrent addition of oubain blocks the transport of Na through the Na/K-ATPase, toxifying the cells and leaving only LoF variants attached. The LoF variants are then collected through fluorescence-activated cell sorting (FACS) and quantified by next-generation sequencing (NGS). This exciting pilot study identified 40 GoF and 33 LoF variants from a library of 224 (228 possible non-synonymous changes) variants. Further, patch clamp analysis validated eight of nine variants helping establish this approach as a powerful functional tool toward assessing the remaining SCN5A VUS. These studies should be considered within the context of the complexity of NaV1.5, β-subunits and other binding partners. Pathogenicity may stem from or be modulated by any of these components and even some of the established “gold standard” techniques should be interpreted with caution (65).

## K^+^ Channels

Potassium channels play important roles in the cardiac action potential including the voltage gated Kv7.1 (KCNQ1) and Kv11.1 (KCNH2) channels, responsible for the outward *I*_*Ks*_ and *I*_*Kr*_ currents during repolarization (Figure 1C). While the inward rectifier Kir2.1, responsible for the outward I_*K*1_ current, also contributes to repolarization, its main function is setting the resting membrane potential (66) (Figure 1C). Unlike Nav1.5 and Cav1.2, these channels are expressed as smaller monomer subunits that combine to form tetramers (Figure 1D). Compared to Nav1.5, LoF variants in Kv7.1 and Kv11.1 are clinically less complex, causing LQT1 and LQT2 (9) respectively, while LoF variants in Kir2.1 cause Anderson-Tawil Syndrome (ATS) and Catecholaminergic Polymorphic Ventricular Tachycardia (CPVT)-phenocopy syndrome (66) (Figure 1D). GoF variants for all three channels also cause short QT syndrome (SQTS). Despite the more straightforward clinical phenotypes and smaller size of these proteins, an incredible >1500 VUS (or conflicting interpretations) are reported in ClinVar for these channels.

### KCNQ1/Kv7.1

Vanoye and co-authors demonstrated the first relatively large K^+^ channel variant study of 78 Kv7.1 variants using CHO-K1 cells. To validate the automated system approach, they measured current density and activation data for 30 pathogenic and benign control variants of homomeric channels, which agreed with manual patch studies (67). With the robustness of the system established, they evaluated 48 test VUS as homomeric channels and 22 LoF co-expressed with WT for comparison (heterozygous-type expression). Of those 22 heteromeric channel recordings, 17 exhibited similar current densities to the homomeric channels and 2 were WT-like. Overall, they provide strong functional data to support reclassification for >65% of variants tested. While no DMS of Kv7.1 have been reported, it is potentially a great candidate for assessing trafficking of each variant in a massively parallel fashion as demonstrated for Kv11.1 below.

### KCNH2/Kv11.1

In a similar automated patch study of heteromeric Kv11.1 channels using HEK 293 cells, Ng and co-authors demonstrated that current densities and gating characteristics for 17 variants reported in ClinVar as pathogenic or benign were in perfect agreement. Of 13 test VUS evaluated, all variants had a dominant-negative effect when co-expressed with WT (68). These relatively small proof-of-principle Kv7.1 and Kv11.1 automated patch studies demonstrate that functional evaluation for all Kv7.1, Kv11.1 and Nav1.5 VUSs are conceivable in the near future.

Measuring current densities is the gold standard for determining ion channel function but proxy assays to investigate mechanism are also important. A good example of this is LQT2, where multiple Kv11.1 LoF mechanisms have been described (69, 70). However, the vast majority (~90%) are trafficking defective, which was shown for over 160 variants using glycosylation differences. However, glycosylation assessed by Western blot is relatively low throughput and a proxy for surface expression (71). A higher-throughput flow cytometry-based method directly measuring cell surface expression was reported with comparable results, but, importantly, some WT-like glycosylation patterns showed reduced trafficking (72). Another advantage is that this assay is multiplexable as recently demonstrated by Kozek and authors, thus can assess surface expression of all possible Kv11.1 variants simultaneously (73). As a pilot study, they targeted 11 residues in the S5 helix, which generated trafficking scores for 220 missense variants. Kv11.1 LoF were then validated for many of these variants with reduced surface expression using patch clamp. In another study, the same flow-cytometry-based DMS analysis targeting 77 residues of the Kv11.1 PAS domain was performed (74). In combination with automated patch-clamp studies, they found variable levels of trafficking with ~40% causing LoF (< 50% of WT) and most were dominant negative, also validated by patch clamp analysis.

Finally, loss of protein stability and misfolding is a major driver of disease (75) and underpins most LoF Kv11.1 variants (71). Developing HTP methods for assessing protein stability in the context of VUS could therefore have great utility in providing pathogenic support (PS3) per ACMG guidelines (Figure 3). A good example of this is Kv11.1, which contains well-characterized intracellular Per-Arnt-Sim (PASD) and cyclic nucleotide-binding homology (CNBD) domains (Figure 1D). We recently reported on the solubility for over 50 recombinantly expressed PASD variants as a proxy for stability and found that it correlated with trafficking data (76). This provides proof-of-principle of DMS to assess protein domain stability and could play an important role in VUS assessment. Indeed, a DMS called VAMP was recently developed like the cell surface DMS described above (Figure 4). This uses a GFP-fusion to quantify total cellular abundance of variants by FACS analysis (77), which could be modified with a GFP folding-reporter system, where GFP fluorescence is linked to the stability of upstream proteins (78). This type of assay could have wide ranging applications particularly for complex multidomain proteins like lamin A/C and titin discussed below.

### KCNJ2/Kir2.1

A recent, massively parallel insertional mutagenesis method was used to determine surface expression for over 300,0000 recombination Kir2.1 variants (79). However, only rarely are KCNJ2 pathogenic variants associated with trafficking defects (80) and alternative DMS approaches for measuring Kir2.1 might be better suited. One possible DMS approach is to adapt yeast-based functional assays that demonstrate normal and LoF variants based on growth patterns, which have been reported for ATS-associated Kir2.1 (73) and Barter Syndrome-associated Kir1.1 (a Kir2.1 paralog that is not arrhythmia related) (81). Regardless, development of a potassium channel DMS is needed for this important arrhythmogenic and underrepresented DMS data group.

## Ca^2+^ Handling Proteins

Another group of proteins underlying inherited cardiac disease are calcium handling proteins, which work in conjunction with the ion currents discussed above to control cardiac contraction and relaxation. Briefly, calcium handling is initiated with Ca_v_1.2, the pore forming alpha subunit of a multimeric ion channel complex, which passes inward L-type calcium current (*I*_*Ca*−*L*_) (Figure 1C). The increase in intracellular calcium promotes further release of calcium from the sarcoplasmic reticulum via ryanodine receptors and both proteins are tightly regulated by the calcium sensor calmodulin (CaM) and calsequstrin (CASQ2) (Figure 1B). Various inherited arrhythmia syndromes have been described due to disturbances in calcium homeostasis (82). For example, LoF CaM and CASQ2 variants have been linked to LQTS and CPVT, respectively. LoF and GoF Ca_v_1.2 (CACNA1C) variants have been associated with Brugada Syndrome and Timothy Syndrome, respectively. GoF ryanodine receptor 2 (RYR2) variants have been linked to CPVT, while LoF RYR2 variants have been linked to CRDS (Calcium Release Deficiency Syndrome) (Figure 1B). Combined, ClinVar reports >3400 VUS or with conflicting interpretations for these calcium handling proteins (Figure 2A). While HTP functional analysis is lacking for CASQ2 and Cav1.2, numerous functional assays have been described for CASQ2 (83) and automated patch clamp has been reported for pharmacological screening of Cav1.2 using HEK 293 cells. While this latter system should be adaptable for HTP functional analysis, Cav1.2 is more complex requiring its two subunits and an inward rectifier for optimization (84). This is just one example of the challenges in developing HTP functional analysis for some proteins, which we discuss further below with RYR2 as well as a yeast-based DMS reported or CaM.

### CALM/Calmodulin

Three genes (CALM1-3) encode the exact same, relatively small Calmodulin (CaM) protein, which contains four EF-hand calcium sensors motifs (Figure 1B). CaM has immense versatility, regulating over 300 targets (85), yet variants have surprisingly low phenotypic complexity showing only cardiac effects (i.e., LQTS) despite its central role in biology (Figure 1D). Cardiac conditions are not surprising however since CaM regulates most cardiac ion channels indirectly or directly including Kv7.1, Nav1.5, Cav1.2 and RyR (86). Variants are located throughout the protein (87) and mechanistically change calcium binding affinity, disrupting critical protein-protein interactions or both, which have been widely reported using a variety of methods (88). Recently, dysfunction of CaM variants has been characterized by exploiting a yeast-based complementation assay where variants of a human gene (e.g., CaM) can be assessed for their ability to rescue a yeast strain carrying a temperature-sensitive allele of the yeast ortholog (e.g., CMD1) (89). In a tour de force, Weile and co-authors performed a DMS of four proteins including CaM generating functional scores for 1813 CaM variants out of 2831 possible (64%) (53). Their assay was also validated showing good agreement between their functional scores and ClinVar annotated pathogenic variants and benign high frequency variants identified from gnomAD. Finally, the authors surveyed the literature for functional assays and found that in addition to the 5% of approximately 4000 human disease genes that already have a yeast complementation assay, 57% have a potential assay that is adaptable for DMS.

### RYR2/Ryanodine Receptor 2

Three genes (RYR1-3) code for ryanodine receptors RyR1-3, which are six transmembrane tetramers but contain uniquely large cytoplasmic regions comprised of many specialized domains including two EF-hand motifs. They make up the largest human channels (>2-MDa) and RyR2 is primarily expressed in cardiac muscle (Figure 1D). However, given their intracellular location and function, they are not amenable for automated patch clamp studies nor the other HTP measurements discussed (90). One attempt used HEK 293 cells stably expressing skeletal muscle RyR1 for time lapse [Ca^2+^]_ER_ measurements in a 96 well format (91). Another used a FRET-based assay that detects changes in binding of RyR accessory proteins (e.g., CaM and FKBP12.6) (92). While these assays are more complex, there is a lot of room for creativity and should be adaptable for HTP RyR2 variant screening.

Perhaps the biggest advance in assessing RyR2 variants is the impressive number of high-resolution crystal structures of individual RyR domains (93, 94) and advances in resolution of full-length cryoEM structures (95). These have permitted extraordinary, detailed insights into variants effects on structure and function for these highly complex proteins (96) and should help guide domain specific HTP assays for assessing variant effects (97). With over 2200 VUS reported in ClinVar (Figure 2A), innovative functional approaches are needed to help close the VUS burden.

## Filamentous Proteins

Filamentous proteins, critical for cellular mechanics and a host of other functions, have been implicated in a variety of inherited arrhythmias. Among these is lamin A/C (LMNA), the most pleiotropic human gene with ~500 VUS causing over a dozen distinct clinical phenotypes including dilated cardiomyopathy (DCM) (98). Additionally, the sarcomeric proteins titin (TTN) contains over 7,000 VUS implicated in 25% of all inherited DCM cases and myosin-7 (MYH7) contains ~1500 VUS associated with HCM and RCM (Figures 1A, 2A) (99). These proteins highlight both the challenges as well as prospective solutions for HTP functional characterization of highly complex multidomain proteins (Figure 1D).

### LMNA/Lamin A/C

The nuclear membrane bilayer is embedded with numerous integral membrane proteins that interact with the nuclear lamina composed of intermediate filament protein lamins A/C, B1 and B2 encoded by LMNA, LMNB1 and LMNB2, respectively (98) (Figure 1B). Functionally, lamins help link the nucleus to the cytoplasm, contribute to nuclear architecture, chromatin organization, regulation of transcription and others (98). Laminopathic variants, spread across its structure consisting of four coiled-coil domains (CCD) and an Ig-like domain (IgD), can cause several different nuclear envelope abnormalities including honeycomb like shapes, blebs and lamin A foci or aggregation in the nucleoplasm (100) (Figure 1D). Given the lamina's pleiotropic functions, an all-encompassing functional assay like patch clamp discussed above for ion channels is not possible and alternative methods are needed.

Our lab recently used a functional genomics approach for over 170 lamin A variants and found that the majority of myopathic variants aggregate, including DCM, using two overexpression models, and validated in iPS-CMs (104). This relatively simple and robust assay could be used to support pathogenicity (PP3) of over half of all DCM linked LMNA variants per ACMG guidelines (Figure 3). Further, many different phenotypes can be analyzed by high content imaging (101) including aggregation, which can also be sorted by FACS (102) enabling DMS as a possibility for lamin aggregation and potentially other cardiomyopathy phenotypes. Alternatively, yeast-based assays have also been developed for DMS of protein domain aggregation by measuring yeast growth (i.e., cytotoxicity) as a correlative proxy of aggregation propensity (103). The prion domain of TDP-43 associated with amyotrophic lateral sclerosis (ALS) is one recent example that covered >50,000 variants, which could be applied to lamin's CCDs and other aggregation prone arrhythmogenic targets. Finally, we applied the same solubility assay described for the Kv11.1 PASD above to over 50 lamin IgD variants and found, unsurprisingly, that IgD solubility inversely correlated with lamin aggregation making this domain amenable to DMS approaches (77, 104).

### TTN/Titin

Contractile and stretching motions take place in the sarcomere of muscles and is the location where titin, the largest human protein, acts as a molecular spring. It is a multidomain protein containing ~300 small Ig-like (IgD) or fibronectin III-like (FN-3) domains among others (18) (Figure 1D). A recent study using a simple solubility assay (similar to the Kv11.1 PASD and lamin IgD studies discussed above) tested 15 TTN-linked missense variants (3 IgD, 11 FN and 1 kinase domain) and revealed domain destabilization as a common disease mechanism, which also correlated with more rigorous biophysical characterizations (105). With these domains making up ~90% of titin, HTP assays like those described for small soluble domains to assess VUS could cover the vast majority of TTN variants.

### MYH7/β-Cardiac Myosin Heavy Chain and the Limitations to Functional Assays

Also located in the sarcomere is β-cardiac myosin, a molecular motor with ATPase activity that is essential for muscle contraction (Figure 1D). It has several functional domains including actin and ATP-binding domains, a lever arm and HCM-enriched converter and myosin head domains (106). We finish with this multidomain protein as it underscores the limitations often associated with functional assays in assessing VUS (107). First, functional assays need to meet a high level of reliability, as outlined by Brnich and co-authors (37). To measure β-cardiac myosin function, various methods including ATPase and motility *in vitro* assays have been developed with limited success. A recent literature review by a Variant Curation Expert Panel (VCEP) reported numerous problems regarding method standardization, lack of controls, conflicting results, and poor reproducibility (107). Consequently, none of these *in vitro* assays were approved as functional evidence at any level to assess their clinical significance leaving only knock-in mouse models to functionally interpret variants. As another example, we reported that for some lamin A variants, aggregation propensity can vary greatly between cell models (104).

Second, LOF does not necessarily prove causality. A recent sequencing study of over 13,000 asymptomatic individuals >70 years old with no history of cardiovascular disease had variants classified as pathogenic in MYH7 as well as most of the other genes described here (e.g.,TTN, RYR2, KCNQ1, SCN5A) (108). This suggests that these variants are either not implicated in disease, or (more likely) points to the polygenic nature for these diseases (Figure 2B). Related, the importance of genetic modifiers is increasingly being recognized toward understanding inherited cardiac disease. For example, a polymorphism in the coding region of SCN5A was reported to modify expression of a LQT3-linked variant (109). Similarly, variants in noncoding enhancer regions of MYH7 and LMNA were recently identified; the former of which correlated with increased HCM progression (110). These examples highlight how genetic background is important for understanding variant pathogenicity making a major limitation to functional studies as well as how ACMG guidelines handle these genetic modifiers.

Finally, variants can exert their effects through other mechanisms such as β-adrenergic stimulation such as KCNQ1, KCNJ2 (111) and RYR2-associated CPVT (90). For example, truncating TTN variants (the most common variants) showed a reduced response to β-adrenergic stress among other dysfunctions in an iPS-CM model (112). To conclude, most of the assays described here rely on overexpression systems in non-cardiac cells (out of necessity, i.e., yeast-based assay) but these in turn can limit their utility. iPS-CMs however have been widely used to characterize inherited cardiac diseases that largely recapitulate disease cellular phenotypes (42). With advanced genome editing technologies in place like base editing (113) and the ability to physically sort a growing number of cellular phenotypes (114), pooled genetic screening of inherited cardiac disease variants in iPS-CMs is surely on the horizon to help overcome some these limitations.

## Computational and Predictive Data

### Variant Prediction

Computational methods are a developing predictive assessment of genetic variants that complement functional data and fill in where functional data is lacking. A high degree of accuracy remains lacking and computational approaches are still considered relatively weak supporting evidence compared to functional methods per ACMG guidelines (Figure 3) (27). The number of tools, however, are growing with each claiming to be superior to the last and covering each is beyond the scope of this review. There are several excellent reviews of these approaches (36, 115, 116) and we provide here just a brief overview of these tools, discuss their advances and shortcomings and how these integrate with cardiac arrhythmia variants. While the tools described herein primarily cover variants in coding-regions, non-coding VUS are also prevalent in cardiac disease and several *in silico* tools have been developed to assess those, notably the neural network-based splice variant predictor SpliceAI (117). These tools along with reporter assays should help toward pathogenic assessment of splice variants via ACMG guidelines (118).

Some of the early and popular prediction tools are relatively simple and rely on sequence conservation like SIFT (119) or use one or more structure-based metrics like protein stability and location like FoldX or combine sequence and structure metrics like PolyPhen-2 (Figure 4D) (115). For example, changes in the physicochemical property of a variant associates with more severe disease in a study comparing 1300 sodium channel variants to their genetic and clinical characteristics (120). Most of these tools, including PolyPhen-2, use machine learning methods trained using numerous metrics such as stability or pathogenic classification data that can be collected from structure-based databases like ProTherm (121) and ADDRESS (122) or variant pathogenicity databases such as HGMD (123) and ClinVar (17). The results of these programs have been collated in databases such as dbNSFP v.4, which provides 36 deleterious prediction scores for the over 84 million possible missense variants in the human genome (124). This in turn has served as a rich source of data for generating ensemble or meta predictors (Figures 4C,D). For example, more integrative approaches have been developed such as REVEL, which incorporates 18 scores from 13 different tools into an ensemble score with improved prediction capabilities (125). There are limitations to these various methods however including; (1) inflated accuracies due to data that is used for training overlapping with data used for benchmarking (126), (2) lower quality pre-genomAD control data used, (3) the relatively small training set of known pathogenic and benign variants in databases like ClinVar and HGMD, (4) annotated data types used for training is subjective and perhaps unreliable or poor quality, and (5) relatively small functional benchmarking data lacking in diversity (127). These make applying current ACMG guidelines problematic, which requires that “all *in silico* programs tested agree on the prediction.” This non-specific requirement is problematic for the integration of computational data as “PP3/BP4 supporting evidence” (27) (Figure 3) since concordance can depend on which tools are used and how many (128, 129). Developing better predictors and more applicable ACMG guidelines would move this technology closer toward variant interpretation utility.

One measure to improve predictive tools is the use of improved population control data from genomAD. Many tools performed poorly when predicting benign variants and genomAD can help determine frequency cut-offs to filter out disease-causing variants (130). Further, these minor allele frequencies (MAFs) are based on a relatively small control dataset in genomAD and VUS interpretation will continue to improve as more population sequences are added. ClinPred integrates normal population variant frequency data from genomAD with cleaner ClinVar annotations into existing pathogenicity scores, which performed better than other top tools across several metrics (131). 3Cnet, uses neural networks trained using population data (genomAD), conservation data (UniREF) and clinical data (ClinVar) to outperform most popular tools including REVEL, SIFT, and PolyPhen previously discussed (132).

Since annotated variants used for training can be unreliable and is relatively limited, a few tools have been reported that circumvent this issue. For example, the popular tool CADD relies on variants fixed in the population but absent from human ancestors for training (133). More recently EVE, by relying on evolutionary sequence variation across organisms as independent evidence, made predictions comparable to large-scale functional data from DMS datasets discussed above (134). This is a promising development since a recent study testing 46 variant prediction tools against 31 DMS datasets found DMS results to be generally superior to most other prediction tools with some exceptions including REVEL (135). In addition to being useful for benchmarking, DMS will continue to be an important resource for improving prediction tools such as DeMasK (136) and Envision (55). For example, the latter was trained using 21,000 variant measurements from nine DMS datasets, which showed superior predictive performance to other methods when tested on large-scale functional data, which improved when incorporating more DMS data.

As with the functional assays, the tools described are largely designed for variants treated as monogenic disorders, but most inherited cardiac diseases are more complex. Several methods such as VarClass have also been developed that incorporate other factors such as gene networks (137). Ultimately, combining computational methods together to enhance prediction performance may be optimal (128). Machine learning tools that incorporate well curated, disease-specific data have shown promise and we provide several examples for a variety of cardiac genes below.

### Structure Prediction

Besides pathogenic prediction programs, variant assessment can be improved by modeling variant effects on protein structure. These, however, require highly accurate structural models which are mostly lacking for relevant cardiac proteins (e.g., ion channels). Proteins also do not function in isolation and faster, more accurate approaches for modeling protein-protein interactions and complexes are needed (138). Accurately predicting protein structure from sequence has been a long-standing goal with incremental advancements since the first competition between prediction programs was held by the Critical assessment of Protein Structure Predictions (CASP) in 1994. 2020 was the turning point when DeepMind's neural network AlphaFold2 predicted protein structures on par with experimentally determined structures (139). This offers unprecedented opportunities for accurate modeling of any protein with significant advances in protein-protein (140) and protein complex modeling (141) already reported. Structural and functional insights into variants associated with inherited arrhythmias will undoubtedly benefit greatly from these tools in the coming years such as molecular dynamics analyses (142–144). Going forward, these structural models will need to be integrated into sets of interacting proteins for the full scope of structure-function relationships.

### *In silico* Studies of Inherited Cardiac Disease

Pathogenic prediction tools have been applied toward assessing arrhythmogenic cardiac VUS and we briefly discuss several studies that improve upon some of the limitations discussed above. In general, these approaches take a more gene-specific approach since tools can be variable across genes. This was observed from a comparative analysis of 7 tools tested on 312 Kv7.1, Kv11.1 and Nav1.5 variants with *in vitro* or co-segregation studies used for P/B annotations (128) as well as a study of RYR1 using three popular prediction programs SIFT, PolyPhen2 and MutationTaster (145).

One approach is to assess a variety of prediction tools and choose the best (or composite score) and combine that with another metric. For example, a composite score from 7 *in silico* tools was combined with location-based analysis to generate separate evidence-based decision trees for Brugada Syndrome and LQTS variants (146). A similar study combined *in silico* analysis with reported functional data, phenotype data and MAFs to support reclassifying CPVT-linked RYR2 variants (147). Another metric used to combine with the *in-silico* tools has been paralogue annotation shown to have a high positive predictive value for LQTS genes (148). This method works by transferring disease variants across paralogues, genes that have evolved by gene duplication with similar functions, to predict pathogenicity. This method was applied to CPVT (RYR2) and Brugada Syndrome (SCN5A) showing that upwards of one-third of novel-missense variants can be annotated by assessing paralogue variants (149). More recently, paralogue annotation was reported for SCN5A and Cav1.2 using updated control data to compare the predictions of ≥ 12 popular *in silico* tools, identifying MutPred and ClinPred as the best predictors, respectively (150, 151). These were then combined with annotations from 20 SCN5A and Cav1.2 paralogues to support reclassifying 74 SCN5A and 39 Cav1.2 VUS as pathogenic.

Other tools have leveraged large functional datasets toward gene-specific predictors. For example, a Bayesian method was conditioned on various Brugada Syndrome variant attributes curated from over 700 publications to generate SCN5A penetrance probabilities (152). Clerx and co-authors combed the literature and applied machine learning on *I*_*Na*_ data for over 200 variants and 20 variant-associated features (e.g., location, physicochemical properties) to improve predictions (153). Another approach used functional data for 107 Kv7.1 variants curated from the literature to train the neural network Q1VarPred, which had superior performance compared to 8 other general prediction methods (154). For LMNA variants, an unsupervised machine learning method was recently reported that overcomes limitations from inadequate number of benign variants to identify VUS with a high potential for pathogenicity (155). Cardiboost is a disease-specific machine learning classifier that relies on well-curated familial cardiomyopathy and inherited arrhythmias specific data with improved performance over state-of-the-art tools (156). Finally, the flood of variant data being reported needs curation and database developments such as TITINdb (157) will be important resources for variant interpretation.

## Conclusion

The first part of the Genomic Era focused on variant identification. Now, the challenge for the second part of the Genomic Era is for the field of functional genomics to design and implement HTP and DMS assays integrated with computational modeling. Innovation and creative approaches to assist with variant characterization are needed to keep pace with the deluge of identified but clinically unactionable genetic variants.

## Author Contributions

CA: primary writer, data collection, and figure rendition. SM, LR, TK, CJ, and BD: editing, writing, and data interpretation. LE: primary editor and project leader. All authors contributed to the article and approved the submitted version.

## Funding

This project was supported in part by the Gary and Marie Weiner Prof. in Cardiovascular Medicine Research (LE), NHLBI R01 HL139738-01 (LE), NHLBI R01 HL141342-01 (BD PI; LE, and CJ co-I) and Ruth L. Kirschstein F32 HL128091 NRSA postdoctoral fellowship CA.

## Conflict of Interest

The authors declare that the research was conducted in the absence of any commercial or financial relationships that could be construed as a potential conflict of interest.

## Publisher's Note

All claims expressed in this article are solely those of the authors and do not necessarily represent those of their affiliated organizations, or those of the publisher, the editors and the reviewers. Any product that may be evaluated in this article, or claim that may be made by its manufacturer, is not guaranteed or endorsed by the publisher.
